# Analysis of the extent of limbic system changes in multiple sclerosis using FreeSurfer and voxel-based morphometry approaches

**DOI:** 10.1371/journal.pone.0274778

**Published:** 2022-09-22

**Authors:** Amanda Frisosky Abuaf, Samuel R. Bunting, Sara Klein, Timothy Carroll, Jake Carpenter-Thompson, Adil Javed, Veronica Cipriani

**Affiliations:** 1 Department of Neurology, The University of Wisconsin, Madison, WI, United States of America; 2 Department of Psychiatry and Behavioral Neuroscience, The University of Chicago, Chicago, IL, United States of America; 3 Department of Neurology, The University of Chicago, Chicago, IL, United States of America; 4 Department of Radiology, The University of Chicago, Chicago, IL, United States of America; University at Buffalo, UNITED STATES

## Abstract

**Background and purpose:**

The limbic brain is involved in diverse cognitive, emotional, and autonomic functions. Injury of the various parts of the limbic system have been correlated with clinical deficits in MS. The purpose of this study was to comprehensively examine different regions of the subcortical limbic system to assess the extent of damage within this entire system as it may be pertinent in correlating with specific aspects of cognitive and behavioral dysfunction in MS by using a fully automated, unbiased segmentation approach.

**Methods:**

Sixty-seven subjects were included in this study, including 52 with multiple sclerosis (MS) and 15 healthy controls. Only patients with stable MS disease, without any relapses, MRI activity, or disability progression were included. Subcortical limbic system segmentation was performed using the FreeSurfer pipeline ScLimbic, which provides volumes for fornix, mammillary bodies, hypothalamus, septal nuclei, nucleus accumbens, and basal forebrain. Hippocampus and anterior thalamic nuclei were added as additional components of the limbic circuitry, also segmented through FreeSurfer. Whole limbic region mask was generated by combining these structures and used for Voxel-based morphometry (VBM) analysis.

**Results:**

The mean [95% confidence interval] of the total limbic system volume was lower (0.22% [0.21–0.23]) in MS compared to healthy controls (0.27%, [0.25–0.29], *p* < .001). Pairwise comparisons of individual limbic regions between MS and controls was significant in the nucleus accumbens (0.046%, [0.043–0.050] vs. 0.059%, [0.051–0.066], *p* = .005), hypothalamus (0.062%, [0.059–0.065] vs. 0.074%, [0.068–0.081], *p* = .001), basal forebrain (0.038%, [0.036–0.040] vs. 0.047%, [0.042–0.051], *p* = .001), hippocampus (0.47%, [0.45–0.49] vs. 0.53%, [0.49–0.57], *p* = .004), and anterior thalamus (0.077%, [0.072–0.082] vs. 0.093%, [0.084–0.10], *p* = .001) after Bonferroni correction. Volume of several limbic regions was significantly correlated with T2 lesion burden and brain parenchymal fraction (BPF). Multiple regression model showed minimal influence of BPF on limbic brain volume and no influence of other demographic and disease state variables. VBM analysis showed cluster differences in the fornix and anterior thalamic nuclei at threshold *p* < 0.05 after adjusting for covariates but the results were insignificant after family-wise error corrections.

**Conclusions:**

The results show evidence that brain volume loss is fairly extensive in the limbic brain. Given the significance of the limbic system in many disease states including MS, such volumetric analyses can be expanded to studying cognitive and emotional disturbances in larger clinical trials. FreeSurfer ScLimbic pipeline provided an efficient and reliable methodology for examining many of the subcortical structures related to the limbic brain.

## Introduction

Multiple sclerosis (MS) is an inflammatory and neurodegenerative disease of the central nervous system (CNS). Inflammatory disease is best visualized in the white matter. Gray matter pathology is more difficult to examine even with the best optimized MRI protocols. Given the drawbacks of directly visualizing gray matter pathology, atrophy is often used as a secondary marker of gray matter damage in MS. Tissue atrophy over time in various regions of the brain not only results from local lesions but also from remote damage in areas directly or indirectly connected to those regions [1,2].

Although focal motor and sensory deficits are typical features of MS, more subtle features of the disease are progressive memory deficits, depression, and fatigue. The anatomical structures mediating these diffuse symptoms of MS include many regions of the limbic system, which play a central role in memory, emotions, and autonomic functions. Several studies have shown that damage in various components of the limbic system is correlated with such clinical deficits. Thalamic atrophy is notable even at early stages in MS, Radiologically Isolated Syndrome (RIS) and Clinically Isolated Syndrome (CIS), and is correlated with memory dysfunction and emotional impulsivity [3–5]. Similarly, other limbic structures such as the fornix, hippocampus, amygdala, cingulum, and hypothalamus have been implicated in cognitive dysfunction, depression, and fatigue in MS [6–14]. The relationship between the more subtle symptoms of MS and limbic system atrophy requires further elaboration, and validated tools for measuring atrophy in small deep gray matter structures still remains exploratory.

The components of the limbic system were originally described by Papez in 1937 and later revised by MacLean in 1949 as a highly interconnected cortical and subcortical structures linking cognitive processes to emotional states [15,16]. The current concept of the limbic system is based on integrated information from animal studies, *in vivo* MRI tractography, and fMRI studies in humans and includes cortical and subcortical structures along the hippocampal-thalamic, hypothalamic, and ventral forebrain [17]. More recently, an automated segmentation pipeline was developed to demarcate various components of the subcortical limbic system from anatomical T1-weighted images [18]. This fully automated tool has been validated and performs well in detecting atrophy in Alzheimer disease compared to controls, however, it has not yet been applied to studies of MS [18].

Given the significance of the limbic system in neurodegenerative diseases, the aim of this study was twofold: first, comprehensively examine the subcortical limbic system in MS by using a newly developed and validated segmentation pipeline, FreeSurfer ScLimbic; second, assess changes in the limbic brain both at the level of regional volumetric analysis and by voxel-based morphometry (VBM). Some of the structures identified through the FreeSurfer pipeline have not been fully examined in previous studies in MS. Hence, this study will further extend the application of this tool to examining deep gray mater pathology in MS with the intent of broadening its applicability to larger studies involving the limbic brain.

## Methods

### Subjects

Healthy and MS subjects were retrospectively identified from the University of Chicago MS database. Subject data were derived from archival medical and radiological records. Clinical assessments, including the Expanded Disability Status scale (EDSS), were derived from comprehensive neurological exams conducted at each patient visit. For the purposes of these analysis, EDSS was dichotomized into high (>4.5) and low (0–4.0) severity. By dichotomizing the EDSS scores, the variance in the EDSS data is reduced and the statistical power to detect the influence of this covariate on the dependent variable is better preserved, especially in multiple testing procedures.

Only relapsing-remitting MS (RRMS) patients were included in this study, with stable disease defined as no relapses, progression, or new MRI activity in the prior 2 years. All MS patients were taking a disease modifying treatment (DMT) at the time of the study. Controls were drawn from healthy subjects without inflammatory or neurodegenerative disease undergoing MRIs for diagnosis such as headaches or non-specific symptoms. All data were anonymized prior to access for analysis. The study was approved by the Institutional Review Board of the University of Chicago Medical Center under protocol number 15–1042, and since this was a retrospective review of data, consent was waived. This study conformed to the ethical standards of the 1964 Declaration of Helsinki.

### MRI acquisition and processing

All MR scans were obtained from a single 3T Phillips Achieva scanner 16-coil (Philips Medical Systems, Best, The Netherlands). The protocol was: 3D T1-weighted Turbo Field Echo (3DT1TFE) TR = 6.9 ms, TE = 3.7 ms, flip angle = 15˚, voxel size = 1 x 1 x 1 mm3, 170 slices, FOV 224 x 224, matrix 228 x 200. FLAIR images TR = 11,000 ms, TE = 125 ms, TI = 2800 ms, flip angle 90, FOV 224 x 224, and matrix 224 x 217, voxel size 2 x 2 mm 80 slices.

All volumetric analyses were performed using FreeSurfer image processing pipelines as previously described [18,19] (v7.1.1 and 7.0-dev; surfer.nmr.mgh.harvard.edu/fswiki/recon-all; FreeSurfer.net/fswiki/ThalamicNuclei; surfer.nmr.mgh.harvard.edu/fswiki/ScLimbic). From the large number of outputs from the FreeSurfer pipeline, only subcortical structures related to limbic circuitry were selected as final output for analysis. In addition to examining the whole thalamus, anterior thalamic nuclei were specifically selected due to their specific involvement in the mammillothalamic tract of the Papez circuit and their role in spatial, verbal, and visual memory [20–22]. The anterior thalamic nuclear group was defined as the anteroventral, laterodorsal, ventral anterior, and ventral anterior magnocellular group as previously described [19,23]. Paired volumes from FreeSurfer output were summed and standardized as percent of intracranial volume as previously described [24]. For FreeSurfer volumetric analysis, white matter lesion hypointensities were not filled in to make them isointense. FreeSurfer program segments white matter hypointensities separately and filling in these lesions does not influence the gray or white matter segmented volumes as has been previously demonstrated in MS [25].

VBM was performed on the dataset as an alternative method of detecting atrophy in the limbic system, using MATLAB R2020b (Mathworks, Natick, MA) and SPM12 (Wellcome Department of Cognitive Neurology, London). In the SPM pipeline, white matter lesions on MRI scans can lead to misclassifications of voxels during image registration process and therefore effect of these lesions was minimized by lesion filling of T1 hypointensities on all T1-weighted images **[26]**. Automated lesion detection was performed using Lesion Segmentation Tool (**LST) in SPM12 (**www.statistical-modelling.de/lst.html). T2-FLAIR scans were coregistered to 3DT1-weighted images and white matter lesions were extracted using the lesion prediction algorithm (LPA) [27,28]. The resulting lesion probability maps were then used to replace T1 hypointense lesions on T1-weighted scans with normal-appearing white matter intensities, i.e. lesion filling [25,29]. Accuracy of the process was confirmed by visual inspection of all images. The modified 3DT1-weighted images were then segmented into GM, WM, and CSF tissue classes, aligned using DARTEL, and normalized to MNI space with 1.5mm cubic resolution [30]. GM images were smoothed using an 8mm full-width at half-maximum (FWHM) isotropic Gaussian kernel. The limbic region mask for VBM was generated using FreeSurfer. One of the control volumes was registered to the MNI template in the same manner as the entire cohort and segmented using the ScLimbic pipeline. The resulting individual regions were binarized to generate masks using a threshold value of 0.2 and then combined to create the final ROI mask consisting of hippocampus, fornix, mammillary bodies, anterior thalamic nuclei, hypothalamus, nucleus accumbens, basal forebrain, and septal nuclei. This was then used as an explicit mask in factorial design specification in SPM.

### Statistical analysis

Demographic data between MS patients and the control group were compared using Fisher’s Exact Tests for categorical and Mann-Whitney *U* tests for continuous variables. Analysis of covariance (ANCOVA) model was used to compare the volumes of the total limbic region and individual subregions between MS and the control groups. Analyses were adjusted for race, sex, age, and body mass index (BMI). The Bonferroni correction was applied to account for multiple comparisons (α = .05/8 = 0.00625). Spearman’s correlation coefficient was used to examine relationship between limbic system atrophy and overall MRI disease burden. Multiple linear regression model was used to assess the effects of overall brain atrophy (BPF) on total limbic volume in MS patients when controlling for disease severity (EDSS), duration of disease, and duration of treatment. Regression models were run twice, once without demographic covariates and a second time adjusting for demographic variables. All volumetric analyses were conducted using Stata V17 (StataCorp, College Station, TX).

For VBM analysis, comparisons between controls and MS groups were performed using t-contrasts in the design matrix within the GLM framework [30,31]. Given the VBM analysis was restricted to a fairly small region of the brain (subcortical limbic ROI) and *a priori* assumption that only limited voxel clusters would be expected to be significant anyway, the statistical comparisons were relaxed using a voxel-wise threshold of *p* < 0.05 between groups, with no correction for multiple comparisons such as family-wise error (FWE) correction. All k clusters > 0 threshold were examined. VBM results were corrected for total intracranial volume (TIV), age, sex, race, and BPF, which was included to account for the influence of whole brain atrophy on the subcortical limbic region of interest.

## Results

### Demographics

A total of 67 subjects were included in this study, including 52 with MS and 15 health controls. Demographic data are shown in Table 1. The median age of the controls was slightly lower 34 (IQR 26–45) compared to MS patients: 43.5 years (IQR 36–49; *p* = .01). Age was the only demographic variable that differed significantly between the two groups, and as such, it was included as a covariate in all subsequent, relevant analyses to control for this difference.

**Table 1:** Baseline demographic, clinical, and MRI characteristics of subjects.

	Control	MS	*P*
	(*n* = 15)	(*n* = 52)	
**Sex**	*N*	*n*	0.99
Female	12	41	
Male	3	11	
**Race**			0.38
White	10	26	
Black	5	26	
Age, years (median, IQR)	34.7 (26–45)	42.7 (36–49)	**0.01**
Baseline EDSS (median, IQR)	NA	2.5 (1.75–5.25)	-
Lower EDSS (≤ 4.0)		34	
High EDSS (>4.5)		18	
Disease Duration, years (Median, IQR)	NA	8.5 (5–15)	-
Treatment Duration, years (median, IQR)	NA	5 (3–8)	-
T2 lesion Volume cm^3^ (Median, IQR)	NA	9.66 (2.93–22.96)	-
BPF (Median, IQR)	0.82 (0.81–0.84)	0.77 (0.73–0.81)	**< .001**
BMI (Median, IQR)	26 (22.1–32.1)	25.7 (23.2–30.7)	0.75

Values represent the demographic characteristics of both the sample of MS patients and healthy control patients. Comparisons of categorical variables were conducted using Fisher’s Exact Test and median values were compared using Mann-Whitney *U*-tests where applicable.

Among the MS patients, median EDSS at baseline was 2.5 (IQR: 1.75–5.25), 34.6% (*n* = 18) were categorized as high disease severity and 34 (65.4%) had low disease severity based on EDSS. Median MS disease duration was 8.5 years (IQR: 5–15), and median treatment duration was 5 years (IQR 3–8), which corresponded to a median of 12.9% (IQR: 6.71–20.5) of years of life, of which 83.3% (IQR: 29.2–90.9) of those years were under treatment. The median T2 lesion volume was 9.66 cm^3^ (IQR: 2.93–22.7). Median BPF values were significantly lower in the MS group than controls (0.77, IQR:[0.73–0.81] vs. 0.82, IQR:[0.81–0.84], *p* = .001).

### Limbic system nuclei atrophy

**Fig 1:**
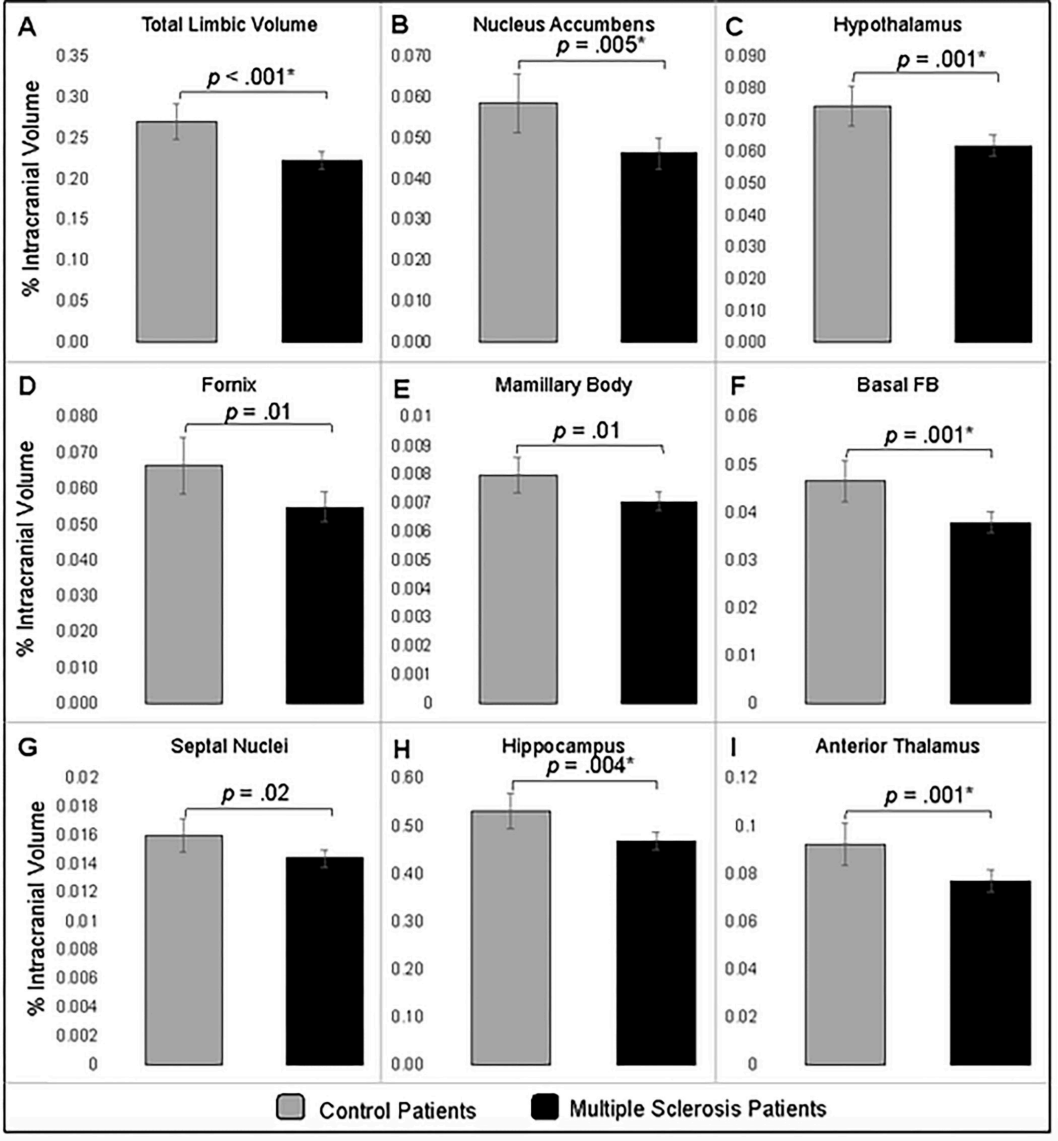
Comparison of limbic region volume between MS and control patients. The mean (95% CI) percentage of total intracranial volume occupied by limbic nuclei that were significantly different between the groups. ANCOVA analyses adjusted for patient race, age, sex, and BMI. The *p*-value for each comparison are presented and an asterisk (*) indicates the comparison remained statistically significant following application of the Bonferroni correction.

The mean [95% confidence interval] of the total limbic system volume was lower (0.22%, [0.21–0.23]) among patients with MS as compared to healthy control patients (0.27%, [0.25–0.29], *p* < .001) (Fig 1A). All limbic subregions were smaller among MS patients as compared to the control patients at the *p* < .05 level. After applying the Bonferroni correction for multiple comparisons, five regions remained significantly different between MS and control patients (Fig 1B–1I): nucleus accumbens (0.046%, [0.043–0.050] vs. 0.059%, [0.051–0.066], *p* = .005), hypothalamus (0.062%, [0.059–0.065] vs. 0.074%, [0.068–0.081], *p* = .001), basal forebrain (0.038%, [0.036–0.040] vs. 0.047%, [0.042–0.051], *p* = .001), hippocampus (0.47%, [0.45–0.49]) vs. 0.53%, [0.49–0.57], *p* = .004), and anterior thalamus (0.077%, [0.072–0.082] vs. 0.093%, [0.084–0.10], *p* = .001).

### Correlation of limbic brain volume with overall disease burden

The volume of individual limbic regions was negatively correlated with T2 lesion burden with the exception of the septal nuclei (all ρ < -0.35, *p* < .05) Conversely, many of the individual limbic regions had a positive correlation with BPF (all ρ > 0.35, *p* < .05) (Table 2). In regression analyses comprising of only the MS cohort, higher BPF was associated with greater limbic region volume (β = 0.26, [0.03–0.49], *p* = .03), and no effects of disease severity (EDSS), treatment duration or disease duration were identified in the unadjusted model (Table 3). After adjusting for patient demographics (BMI, age, sex, race), higher BPF was still associated with greater limbic system volume (β = 0.26, [0.00–0.51], *p* = .047) and no significant effects of patient demographics nor disease indices were identified in the fully adjusted model. The final regression model explained 21.4% of the variation in total limbic volume (adj. *r*^2^ = 0.21).

**Table 2:** Spearman correlation coefficients between T2 Lesion volume or BPF and individual limbic regions or the total area.

	T2 Lesion Volume	BPF
Nucleus Accumbens	-0.56***	0.42**
Hypothalamus	-0.55***	0.52***
Fornix	-0.35*	0.25
Basal Forebrain	-0.49***	0.38**
Mammillary Bodies	-0.32*	0.20
Septal Nucleus	0.07	-0.16
Hippocampus	-0.51***	0.52***
Anterior Thalamus	-0.70***	0.73***
Total Limbic Volume	-0.54***	0.42**

* *p* < .05

** *p* < .01

*** *p* < .001.

**Table 3:** Unadjusted and adjusted regression analysis in MS patients.

	Unadjusted				Adjusted			
	*β*	95%CI		*p*	*β*	95%CI		*p*
BPF	0.26	(0.03,	0.49)	**0.03**	0.26	(0.00,	0.51)	**0.047**
EDSS—High Severity (Low)	-0.02	(-0.04,	0.01)	0.20	-0.02	(-0.05,	0.01)	0.15
Duration MS	-0.04	(-0.17,	0.08)	0.52	0.00	(-0.13,	0.13)	0.98
Duration Treatment	-0.01	(-0.05,	0.03)	0.49	-0.02	(-0.06,	0.02)	0.27
Age (years)					0.00	(0.00,	0.00)	0.42
BMI					0.00	(0.00,	0.00)	0.38
Sex—Male (Female)					-0.03	(-0.06,	0.00)	0.06
Race—Black (White)					0.00	(-0.03,	0.02)	0.81

Analyses were run twice, the first model contained only MS disease characteristics and the second model adjusted for patient demographic characteristics (*n* = 52).

### Voxel-wise analyses

**Fig 2:**
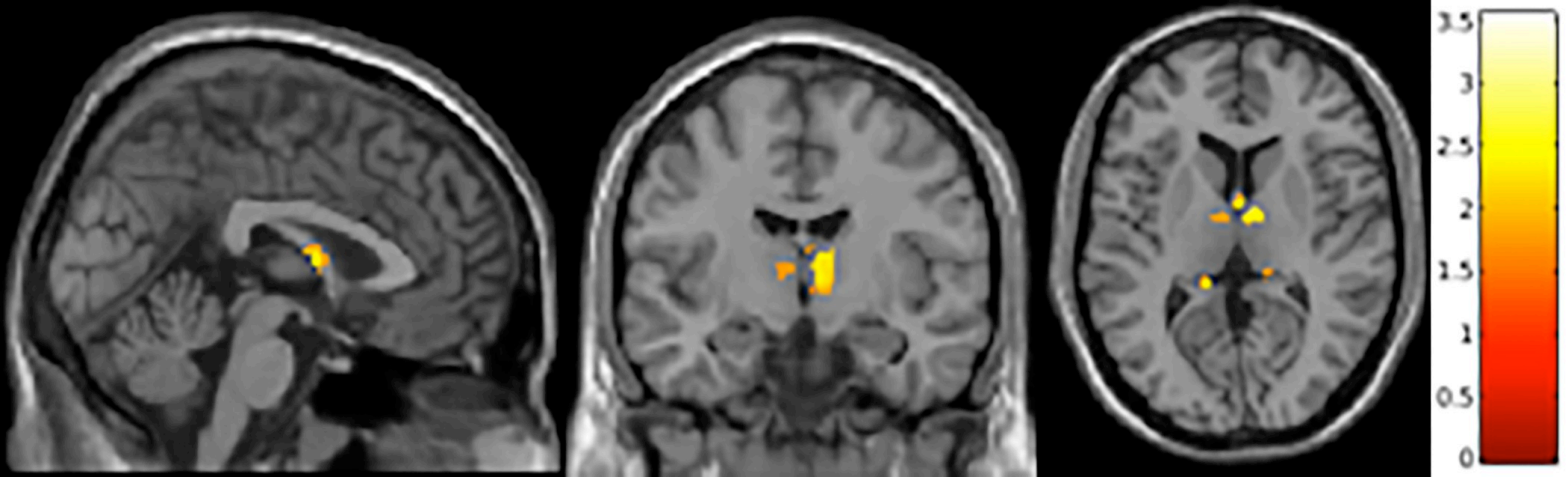
VBM analysis of limbic regions in MS. Fornix and anterior nuclei group are atrophied compared to controls, voxel-wise threshold *p* < 0.05 (uncorrected) and extent threshold K >0, adjusted for covariates TIV, age, sex, race, and BPF.

Regional gray matter changes between controls and MS patients were examined through VBM (Fig 2). When height threshold (voxel level) was set to *p* < 0.05, familywise error uncorrected, and extend threshold K >0, clusters that showed difference between controls and MS included those in the fornix and anterior thalamic nuclei. However, these clusters were not significant after familywise error was set to p < 0.05. VBM analysis was adjusted for total intracranial volume (TIV), age, sex, race, and BPF.

## Discussion

Alterations in the limbic system in MS have been previously demonstrated in several studies using different methodologies. Using volumetric analyses, atrophy in the thalamus, hippocampus, and amygdala have been described [3,32–34]. VBM studies have shown changes in the insula, orbitofrontal cortices, temporal lobes, thalamus, and cingulate cortex [35]. Using diffusion tensor and tractography, the hippocampus, fornix, and thalamus show significant difference from controls [36,37]. Functional MRIs studies highlighted alterations in hippocampal and amygdaloid regions [38]. This study extends previous findings to include most regions of the limbic brain, such as hippocampus, fornix, mammillary bodies, specific thalamic nuclei, hypothalamus, septal nuclei, nucleus accumbens, and basal forebrain, an approach that is more comprehensive than has previously been explored.

The results of this study show that many of the subcortical limbic structures are correlated with measures of diffuse disease burden, such as T2 lesion volume and BPF. A high disease burden suggests a greater probability of extensive disruption of pathways among these highly interconnected brain structures, thereby explaining such a degree of correlation. During the regression analysis, after adjusting for the demographic and disease severity indices, BPF still had a significant influence on limbic region atrophy, *p* = 0.047 (Table 3). Similar analysis was performed using normalized brain volumes, total brain and individual limbic structures. As predicted by using the ratio data, regression analysis using normalized volumes also showed that limbic atrophy was significantly affected by global volume loss (S1 Table). Hence, limbic system atrophy in MS is closely tied to global disease severity in MS. Nonetheless, there could be a smaller effect on atrophy from local tissue pathology, as supported by observations of lesions and neuronal loss in subcortical gray matter structures using histological or ultra-high-field 7T [39–42].

The methodology used in this study to segment the limbic brain also differs from prior studies. In previous studies, segmentation of various subcortical structures has relied on generation of masks either manually or by deforming labels from atlases to a subject through nonlinear registration methods [18]. Such methods are either too time consuming in terms of manual masks or limited to few structures based on the choice of atlas used. The FreeSurfer pipeline offers the advantage of segmenting many subcortical limbic structures (nucleus accumbens, basal forebrain, septal nuclei, hypothalamus, mammillary bodies, and fornix) at the same time using only T1-weighted MRI scans with good reliability and precision [18]. For dissection of the subcortical brain, the FreeSurfer uses a U-Net architecture that is trained on 39 manually labeled MRI datasets and employs spatial, intensity, contrast, and noise augmentation parameters to yield precise segmentations [18]. The tool shows good test-retest reliability. Of note, many of the volumes segmented using this pipeline showed high correlation with atrophy in Alzheimer’s disease or due to aging [18] but in this study, this methodology is further extended to MS disease state.

Two different methodologies were used to demonstrate changes in the limbic brain, volumetric and voxel-based analyses. The goal was not necessarily to compare the performance of these two techniques but to evaluate the feasibility and sensitivity of these methods in detecting changes in the limbic brain in MS. Pairwise comparison of several limbic volumes showed a significant difference in MS from controls even after adjusting for multiple comparisons. Such volumetric analyses are a fairly standard way of evaluating brain atrophy in diverse disease states. VBM is used to examine the ‘density’ or amount of gray matter present in a given region or voxels between two groups [43]. VBM may also be a more efficient method than ROI analysis for comparing groups of subjects across different gray matter structures all at once. VBM analysis herein detected voxel clusters in the fornix and anterior thalamic nuclei that were significantly different from controls at *p*<0.05 threshold. However, when multiple comparison correction using family-wise error (FWE) was applied to these voxels, the results were not significant between the groups. It should be noted that in VBM, a large number of comparisons are made between hundreds or even thousands of voxels. A very stringent approach is taken for multiple comparison through FWE to control for type 1 errors. Inherent in this approach is the possibility of committing type II error. In dealing with small regions of the brain such as the subcortical limbic brain, occupying < 0.5% of the total brain volume, only few clusters are expected to have significantly different morphometry between groups and further adjusting the alpha value at a higher threshold could overestimate type I error. This may be even more relevant when the sample and effect size are small as well as multiple covariate adjustments are performed in the model. Hence, VBM analysis in this study had lower statistical constraints and were performed more for exploratory reasons and feasibility for future studies. It appears from the results herein that regional ROI volumetric assessment may be more feasible for small brain volumes than VBM.

Clinical studies in MS often use brain atrophy and changes in cognitive scales to assess disease progression and therapeutic efficacy of disease modifying therapies (DMT). Cognitive scales such as Symbol Digit Modalities Test (SDMT), Paced Auditory Serial Addition Test (PASAT), Brief Repeatable Neuropsychological Battery (5 tests), Minimal Assessment of Cognitive Function in MS (7 tests), Brief International Cognitive Assessment for MS (BICAMS), and more recently NIH Toolbox are typically used to examine cognitive dysfunction in MS [44,45]. Atrophy in brain regions is often correlated with changes in the cognitive scales to implicate their role in mediating clinical deficits. Alterations in the deep gray matter specifically related to the limbic brain may provide better assessment of other aspects of dysfunction in MS, such as depression, anxiety, fatigue, anhedonia, frustration, anger, sleep, or arousal. Scales to assess these behavioral functions are available and their use in relation to the limbic brain may provide better assessment of emotional and autonomic imbalances in MS [46–50]. FreeSurfer methodology may provide an easily accessible tool for segmenting limbic brain and its use could be adapted in larger clinical trials to focus not only on cognitive but also emotional aspects of MS disease. Furthermore, as a reliable segmentation tool, this approach raises the possibility that it could function as a useful method of assessing atrophy in early MS and allow clinicians to better predict disease progression.

This study has certain limitations, including a small sample size, retrospective analysis, cross-sectional design. Despite these limitations, the results of this study in MS do provide clear evidence that brain volume loss occurs in many regions of the limbic brain, most significantly in the hippocampus, anterior thalamus, hypothalamus, nucleus accumbens, and basal forebrain. Future prospective trials with a larger sample size and with appropriate cognitive and emotional behavioral scales would provide a more robust and specific analysis of the limbic brain dysfunction in MS. This study also provides usefulness of the FreeSurfer limbic pipeline in assessing limbic system damage in multiple sclerosis, and its use can be expanded to larger clinical trials.

## Supporting information

S1 Table Unadjusted and adjusted regression analysis in MS patients using normalized total and limbic brain volumes. (DOCX)
