# Supplementary material for: Analysis of the extent of limbic system changes in multiple sclerosis using FreeSurfer and voxel-based morphometry approaches
Source: PLoS One. 2022 Sep 22;17(9):e0274778. doi: 10.1371/journal.pone.0274778 (PMC9499213; doi:10.1371/journal.pone.0274778)
Supplement: S1 Table — (DOCX) [file pone.0274778.s001.docx]

**Table** 3 (Supplement): Unadjusted and adjusted Regression analysis in MS patients using normalized total and limbic brain volumes

|  |  |  |  |  |
| --- | --- | --- | --- | --- |
|  | Unadjusted | | Adjusted | |
|  | *β* (95%CI) | *p* | *β* (95%CI) | *p* |
| Norm. Total Brain Vol. (mm^3^) | 0.003 (0.002, 0.004) | **<.001** | 0.003 0.002, 0.004) | **<.001** |
| EDSS - High Severity (Low) | -83.0 (-306.8, 140.7) | 0.46 | -122.6 (-357.7, 112.5) | 0.30 |
| Duration MS | -570.2 (-1513.9, 373.5) | 0.23 | -333.6 (-1363.4, 696.3) | 0.52 |
| Duration Treatment | -47.3 (-349.3, 254.6) | 0.75 | -101.8 (-419.6, 216.0) | 0.52 |
| Age (years) |  |  | 2.4 (-6.5, 11.3) | 0.59 |
| BMI |  |  | 10.5 (-6.4, 27.4) | 0.22 |
| Sex - Male (Female) |  |  | -98.8 (-332.8, 135.3) | 0.40 |
| Race - Black (White) |  |  | 22.0 (-169.7, 213.8) | 0.82 |

Linear regression analyses predicting normalized, total limbic system volume and normalized total brain volume as the main independent variable. Analyses were run twice, the first model contained only MS disease characteristics and the second model adjusted for patient demographic characteristics (*n* = 52).
